# Correlation of serum fatty acid binding protein-4 and interleukin-6 with airflow limitation and quality of life in stable and acute exacerbation of COPD

**DOI:** 10.3906/sag-1909-9

**Published:** 2020-04-09

**Authors:** Mohammad Reza ASLANI, Zeynab GHAZAEI, Hassan GHOBADI

**Affiliations:** 1 Lung Inflammatory Diseases Research Centre, Faculty of Medicine, Ardabil University of Medical Sciences, Ardabil Iran; 2 Neurogenic Inflammation Research Centre, Mashhad University of Medical Sciences, Mashhad Iran; 3 Faculty of Medicine, Ardabil University of Medical Sciences, Ardabil Iran; 4 Internal Medicine Department (Pulmonary Division), Faculty of Medicine, Ardabil University of Medical Sciences, Ardabil Iran

**Keywords:** Chronic obstructive pulmonary disease, fatty acid binding protein 4, interleukin-6, airflow limitation

## Abstract

**Background/aim:**

The serum fatty acid binding protein 4 (FABP-4) level increases in chronic inflammatory diseases. The present study aimed to examine serum FABP-4 and interleukin (IL)-6 levels in patients with stable and acute exacerbation of chronic obstructive pulmonary disease (COPD) and the correlation of these markers with airflow limitation.

**Materials and methods:**

We measured serum FABP-4 and IL-6 levels in 60 COPD patients [30 stable COPD (SCOPD), and 30 acute exacerbation of COPD (AECOPD)], and 30 healthy subjects and compared them with airflow limitation according to the COPD stage in the Global Initiative for Chronic Obstructive Pulmonary Disease (GOLD) criteria, peripheral O2 saturation (SpO2), and COPD Assessment Test (CAT) score. We also tested the association between serum FABP-4 levels and some characteristics of study parameters.

**Results:**

Both serum FABP-4 and IL-6 levels increased with increasing severity of GOLD grades in SCOPD (P < 0.01 for both) and AECOPD groups (P < 0.001 and P < 0.01, respectively). It also increased in patients with AECOPD group compared with SCOPD group in GOLD grades I-II (P < 0.01) and GOLD grades III-IV (P < 0.05). In addition, there was a significant positive correlation between serum FABP-4 level with IL-6, CAT score, and smoking history and inversely with FEV1 and SpO2.

**Conclusion:**

The study revealed that serum FABP-4 level was elevated with increasing GOLD grades in COPD patients, markedly in acute exacerbation phase. The increase was associated with elevated serum levels of IL-6 and severity of hypoxia. Thus, it seems that FABP-4 may be involved in the pathogenesis of COPD.

## 1. Introduction

Chronic obstructive pulmonary disease (COPD) is one of the diseases that deeply affects morbidity and mortality in the world [1]. The limitation of air flow is the main characteristic of the disease, which is usually associated with airway inflammation [1]. On the other hand, acute exacerbation of COPD (AECOPD) is characterized by increased systemic inflammation, worsening of pulmonary function tests, increased sputum production, worsening of dyspnoea and cough, and reduced health-associated quality of life, all of which affect the patient’s survival [2]. Similar to other diseases that are not characterized by specific aetiology, various factors contribute to the pathogenesis of the disease, including abnormal immune responses, environmental and hormonal factors, and variable gene expression [3]. 

It has been well-known that under the conditions of AECOPD there is severe airway and systemic inflammation [4]. Additionally, systemic inflammatory markers and cytokines such as interleukin-6 (IL-6), IL-8, fibrinogen, α1-antitrypsin, myeloperoxidase, C-reactive protein (CRP), and tumor necrosis factor alpha (TNF-α) are high with COPD in the exacerbation phase [3,4]. Despite extensive evidence, the precise mechanism of systemic inflammation in AECOPD is not completely clear. It has been reported that a variety of markers and cytokines peak during AECOPD. 

Adipokines (also known as adipocytokines) are adipocyte-secreted protein mediators that not only regulate energy metabolism, but they also control inflammatory responses in many chronic diseases [u291a]. FABP-4 promotes the secretion of proinflammatory cytokines such as IL-6 and TNF-α which exacerbate the severity of the inflammatory diseases [13]. On the other hand, it has been shown that by inhibiting the expression of FABP-4 by pharmacological and genetic studies, many inflammatory signals are inhibited, including the production of inducible nitric oxide synthase (iNOS), cyclooxygenase 2 (COX-2), and inflammatory cytokines such as IL-6, IL-1β, TNF-α [14]. In animal studies with an allergic airway inflammation model in FABP-4-knockout mice, reduced levels of cytokines and inflammatory cells in the lung tissue have been shown [15]. In addition, elevated serum levels of FABP-4 have been reported in chronic inflammatory diseases, such as asthma, rheumatoid arthritis, hypertension, insulin resistance, obesity, cardiac dysfunction, type 2 diabetes, atherosclerosis, acute lung injury, and female stable COPD [16]. The present study was conducted to evaluate serum FABP-4 and IL-6 levels in men with stable and acute exacerbation of COPD. Another aim of this study was to evaluate the relationship between serum FABP-4 levels and the severity of disease based on Global Initiative for Chronic Obstructive Pulmonary Disease (GOLD) grades and clinical assessment of disease severity based on COPD Assessment Test (CAT) in patients with COPD.

## 2. Materials and methods

### 2.1. Study participants

In the current study, 90 subjects were divided into 3 groups including 30 patients with stable COPD (SCOPD), 30 patients with acute exacerbation of COPD (AECOPD), and 30 healthy subjects who were enrolled from April 2017 to March 2018. All subjects were male and matched for age. Patients with a diagnosis of SCOPD were recruited from a respiratory clinic and patients with AECOPD were selected from among those admitted to the emergency department of Ardabil Imam Khomeini Educational and Clinical Hospital, Ardabil, Iran. The control group consisted of subjects with normal spirometry with no respiratory symptoms who were selected from the same hospital who visited in other outpatient clinics.

The SCOPD and AECOPD were diagnosed according to the American Thoracic Society (ATS) guidelines [17]. The AECOPD refers to a sudden worsening of COPD symptoms in the form of shortness of breath, chronic worsening of the cough, and increased volume or discoloration of the phlegm. The inclusion criterion for COPD patients was a ratio of the forced expiratory volume in 1 s (FEV1) to forced vital capacity (FVC) < 70%. Exclusion criteria were the history of hospitalization in the previous 4 weeks, cardiac ischemia, chronic inflammatory diseases, infectious disease, diabetes, metabolic syndrome, chronic renal failure, and cancer.

The pulmonary function testing was performed using a spirometer (Chest Inc., 801, Tokyo, Japan) according to ATS guidelines under standard conditions. Pulmonary function and biochemical tests were conducted on the same day for SCOPD and control subjects, but for the AECOPD patients they were carried out one day after admission to the hospital. The GOLD grades and COPD assessment test (CAT) questionnaire were fully completed for all patients described previously [18]. 

### 2.2. Biochemical measurements

Approximately 3–5 mL of blood samples were taken from all patients to determine serum levels of FABP-4 and IL-6. Serum FABP-4 and IL-6 concentrations were measured using a commercial kit (Crystal day, China) and an electrochemiluminescent method with an Elecsys 2010 automated analyser (Roche Diagnostics). The results were reported as ng/mL.

### 2.3. Statistical analysis

The results are presented as mean ± standard deviation (SD), or median and 25th–75th percentiles. Continuous variables were compared using the student’s t-test. Comparison between groups was made by ANOVA test was performed with Tukey Kemar post hoc test; alternatively, the Kruskal–Wallis test; if significant, it was followed by the Mann–Whitney U* test* for post hoc analysis. Correlation coefficients were assessed using the Pearson’s (or Spearman rank order) correlation test. Linear regression analyses were performed using FABP-4 as dependent variable and IL-6, FEV1, cigarette history (pack/year), GOLD grade, and Spo2 as independent variables. A value of P < 0.05 was considered significant. SPSS version 16.0 (SPSS Inc., Chicago, IL, USA) and Graph Pad Prism 5 software were used for statistical analysis. 

## 3. Results

### 3.1. Baseline characteristics of study population

The study population consisted of 90 men, comprising 30 control subjects, 30 patients with SCOPD, and 30 patients with AECOPD. In the control group the mean age was 58.13 ± 7.36 years and that of the COPD group was 58.70 ± 8.01 years (P = 0.730) (Table 1). 

**Table 1:** Baseline characteristics of patients with COPD and control subjects.

Parameters	Control group	COPD group		
	(n = 30)	SCOPD(n = 30)	AECOPD(n = 30)	P-value
Mean age (year)	58.13 ± 7.36	57.97 ± 9.27	59.43 ± 6.59	0.730
Body mass index (kg/m2)	26.29 ± 3.52	26.27 ± 4.83	24.47 ± 4.38	0.174
Pulmonary function test:				
FEV1 (% of predicted)	90.33 ± 7.98	53.56 ± 23.20	34.04 ± 14.01	0.000
FVC (% of predicted)	85.40 ± 9.26	68.20 ± 22.26	49.53 ± 21.72	0.000
FEV1/FVC	86.70 ± 4.21	58.76 ± 9.77	55.08 ± 11.24	0.000
FABP-4 (ng/mL)	0.80 (0.70–0.90)	0.90 (0.80–1.20)	1.10 (1–1.40)	0.000
IL-6 (ng/mL)	53.50 (43–58)	56 (53–61)	90.50 (76–109)	0.000

Data are presented as mean ± SD or median (25th–75th percentiles). AECOPD: acute exacerbation of COPD, SCOPD: stable COPD, FEV1: forced expiratory volume in 1 s, FVC: forced volume capacity, FABP- 4: fatty acid binding protein 4, IL-6: interleukin 6.

**Figure 1:**
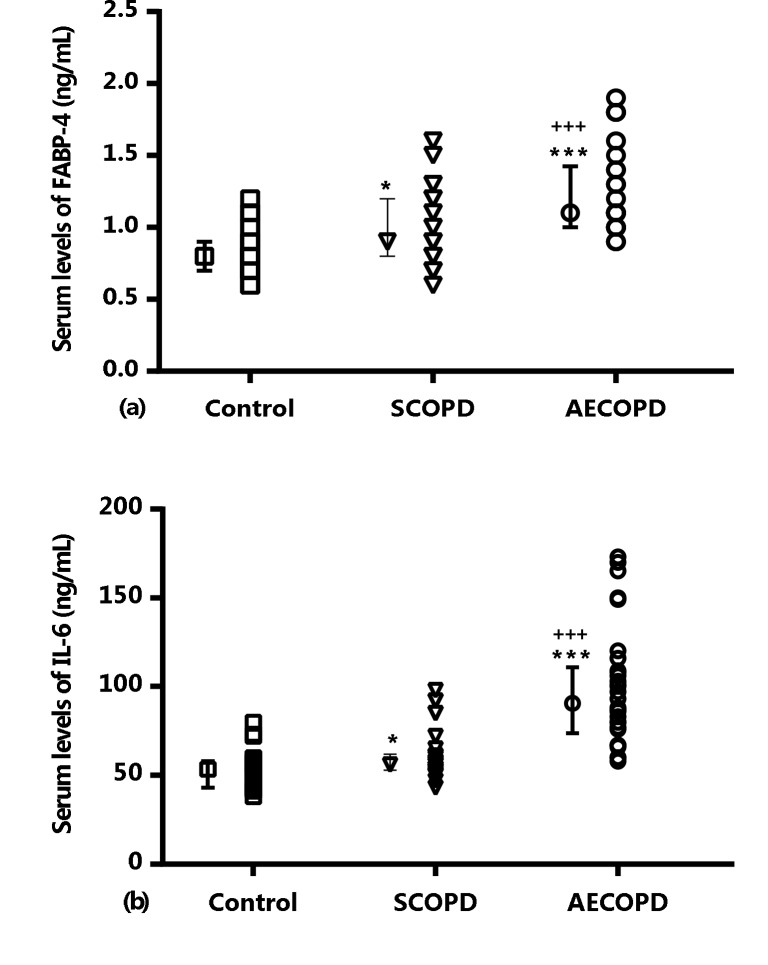
Individual values and median (25th to 75th percentiles) of serum levels of (a): FABP-4 and (b): IL-6 in study groups. For statistical differences between control group and other groups: *; P < 0.05, ***; P < 0.001. For statistical differences between stable COPD with acute exacerbation of COPD: +++; P < 0.001. FABP- 4: fatty acid binding protein 4, IL-6: interleukin-6.

The serum FABP-4 levels were significantly higher in the AECOPD group than in the control and SCOPD groups (P = 0.000 for both, Figure 1a). In addition, there was a significant difference in FABP-4 serum level between SCOPD and control subjects (P = 0.010, Figure 1a). Further, IL-6 results revealed higher levels of IL-6 in AECOPD group than in control and SCOPD groups (P = 0.000 for both, Figure 1b). On the other hand, serum levels of IL-6 in SCOPD group were higher than control subjects (P = 0.023, Figure 1b). 

### 3.2. Severity of chronic obstructive pulmonary disease and baseline characteristics of the study population

Baseline parameters of the study population for COPD severity based on GOLD grade are presented in Table 2. There were statistically significant differences in SpO2 (P = 0.003), smoking history (pack/year) (P = 0.002), CAT score (P = 0.000), FABP-4 level (P = 0.000), and IL-6 (P = 0.000) between the COPD groups based on GOLD grade. However, we found no significant differences in age (P = 0.849) and BMI (P = 0.735) according to the stages of COPD.

**Table 2:** GOLD groups and baseline characteristics of the study population.

Variables	GOLD I-II		GOLD III-IV		SCOPD	AECOPD	SCOPD	AECOPD
Number	14	8	16	22
Age (year)	57.21 ± 9.20	60.50 ± 11.60	58.62 ± 9.59	59.05 ± 3.82
BMI (kg/m2)	25.46 ± 4.06	24.47 ± 4.21	26.98 ± 5.44	24.48 ± 4.53
Smoking (pack per year)P1P2	22 (20–28)	30 (15–58)NS	32.5 (27–39)NS	44.5 (35–78)P < 0.01P < 0.05
FEV1 (% predicted)P1P2	74.78 ± 13.50	52.98 ± 2.50P < 0.001	35.00 ± 9.68P < 0.001	27.15 ± 9.08P < 0.05P < 0.001
FVC (% predicted)P1P2	87.64 ± 14.69	70.75 ± 5.95P < 0.01	51.18 ± 10.48P < 0.001	41.82 ± 20.14NSP < 0.001
FEV1/FVC (%)P1P2	65.00 ± 3.16	58.97 ± 5.62P < 0.01	53.31 ± 10.40P<0.001	53.66 ± 12.49NSNS
SpO2 (%)P1P2	96 (92–96)	89 (85.50–89.50)P < 0.001	92 (89–95)P < 0.05	85.50 (81–88)P < 0.001P < 0.05
CAT scoreP1P2	11 (9–16)	14 (13–15)NS	27 (21.50–31)P < 0.001	26.5 (22–28)NSP < 0.001
IL-6 (ng/mL)P1P2	53.50 (51–56)	67 (63.50–80)P < 0.001	61 (55.50–85)P < 0.01	102 (86–120)P < 0.001P < 0.01
FABP-4 (ng/mL)P1P2	0.80 (0.70–0.90)	1 (0.90–1)P < 0.01	1.10 (0.90–1.25)P < 0.01	1.25 (1.10–1.60)P < 0.05P < 0.001

Data are depicted as mean ± SD or median (25th to 75th percentiles). GOLD: the Global Initiative for Chronic Obstructive Lung Disease, SCOPD: stable of chronic obstructive pulmonary disease, AECOPD: acute exacerbation of chronic obstructive pulmonary disease, BMI: body mass index, FEV1: forced expiratory volume in 1 s, FVC: forced volume capacity, SpO2: O2 saturation, FABP-4: fatty acid binding protein 4, IL-6: interleukin-6, CAT: COPD assessment test, NS: nonsignificant. P1: statistical differences between SCOPD with AECOPD groups. P2: statistical differences between GOLD I-II with GOLD III-IV in study groups.

**Figure 2:**
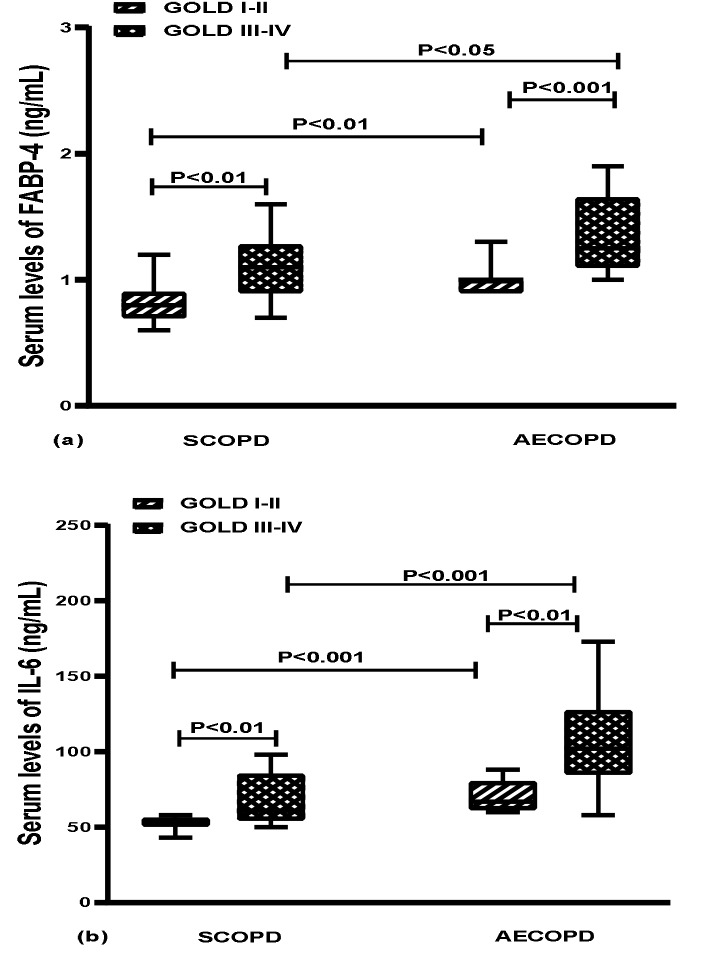
Serum levels of (a): FABP-4 and (b): IL-6 based on GOLD grade in stable and acute exacerbation of COPD patients. Data are depicted as median and quartiles range. GOLD: the Global Initiative for Chronic Obstructive Lung Disease, SCOPD: stable of chronic obstructive pulmonary disease, AECOPD: acute exacerbation of chronic obstructive pulmonary disease, FABP-4: fatty acid binding protein 4, IL-6: interleukin-6.

All grade III-IV patients have higher levels of FABP-4 and IL-6 than the patients with lower grades both in AECOPD and stable groups (P < 0.01 to P < 0.001, Table 2). The AECOPD patients had higher FABP-4 and IL-6 than stable patients in all COPD grades (P < 0.05 and P < 0.001, respectively) (Figure 2a and 2b).

The results showed that in both AECOPD and SCOPD groups, CAT scores were statistically higher in GOLD grades III-IV compared to stages I-II (P = 0.000 for both) (Table 2).

### 3.3. Relationship between serum levels of fatty acid binding protein-4 plus IL-6 and parameters of pulmonary function

**Figure 3:**
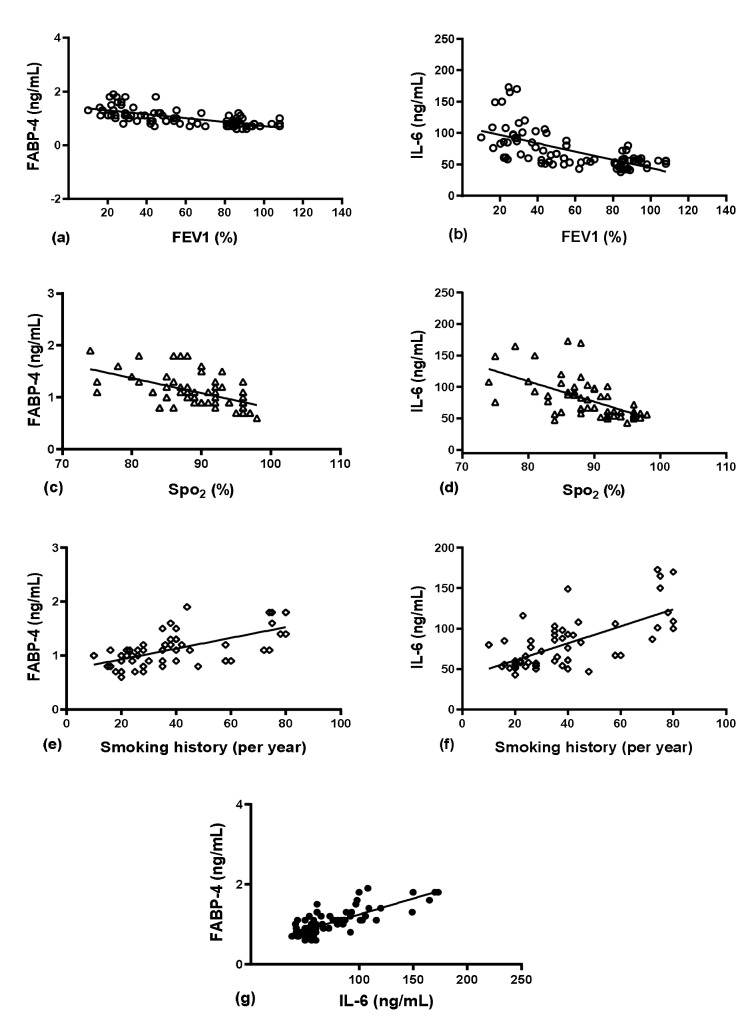
Spearman rank order correlation analysis of (a): FEV1 and FABP-4 serum levels (correlation coefficient = –0.716, P = 0.000), (b): smoking history and FABP-4 serum levels (correlation coefficient = 0.619, P = 0.000), (c): O2 saturation and FABP-4 serum levels (correlation coefficient = –0.537, P = 0.000), (d): FEV1 and IL-6 serum levels (correlation coefficient = –0.666, P = 0.000), (e): smoking history and IL-6 serum levels (correlation coefficient = 0.644, P = 0.000), (f): O2 saturation and IL-6 serum levels (correlation coefficient = –0.640, P = 0.000), and (g): serum levels of FABP-4 and IL-6 (correlation coefficient = 0.692, P = 0.000). FEV1: forced expiratory volume in 1 s, FABP-4: fatty acid binding protein 4, IL-6: interleukin-6, SpO2: O2-saturation.

With regard to study parameters, serum FABP-4 levels were significantly associated with FEV1% predicted (r = –0.716, P = 0.000; Figure 3a), FVC% predicted (r = –0.670, P = 0.000), GOLD grades (r = 0.603, P = 0.000), and smoking history (pack/year) (r = 0.619, P = 0.000; Figure 3b). There were also significant correlations between serum FABP-4 levels and CAT score (r = 0.639, P = 0.000), and SpO2 (r = –0.537, P = 0.000; Figure 3c) (Table 3).

**Table 3:** Spearman correlation analysis of study parameters with FABP-4 in COPD patients (n = 60)

Variables	FABP-4	
	R	P-value
Age	0.069	0.599
Smoking history (per year)	0.608	0.000
FEV1 (% predicted)	–0.672	0.000
FVC (% predicted)	–0.651	0.000
FEV1/FVC	–0.242	0.060
GOLD grade	0.603	0.000
SpO_2_	–0.537	0.000
CAT score	0.693	0.000

FEV1: forced expiratory volume in 1 s, FVC: forced volume capacity, FABP-4: fatty acid binding protein 4, IL-6: interleukin 6, SpO_2_: O2 saturation, GOLD: the Global Initiative for Chronic Obstructive Lung Disease, CAT: COPD assessment test.

Further, the results showed that there were significant associations between IL-6 and FEV1% predicted (r = –0.666, P = 0.000; Figure 3d), smoking history (pack/year) (r = 0.644, P = 0.000; Figure 3e), and SpO2 (r = –0.640, P = 0.000; Figure 3f). Interestingly, there were also significant correlations between serum levels of FABP-4 and IL-6 (r = 0.692, P = 0.000; Figure g).

Finally, a multiple regression was run to predict FABP-4 from IL-6, CAT score, and Spo2. These variables significantly predicted FABP-4, with F (4, 55) = 30.88, P < 0.001, R2 = 0.692. The results showed that the most significant predictors of FABP-4 were IL-6 and CAT score (P < 0.01 and P < 0.001, respectively) (Table 4).

**Table 4:** Associations between FABP-4 and study parameters.

FABP-4			
Variables	B	95% CI for B	P-value
Spo_2_	–0.059	–0.013–0.007	0.538
IL-6	0.420	0.001–0.002	0.001
CAT score	0.361	0.007–0.021	0.000
Cigarette smoking	0.172	–0.000–0.006	0.093

B: unstandardized coefficient. CI: confidence intervals, FABP-4: fatty acid binding protein 4, IL-6: interleukin-6, SpO_2_: O_2_ saturation.

## 4. Discussion

In this study, serum FABP-4 and IL-6 levels were significantly elevated with disease severity based on GOLD grades in patients with stable and acute exacerbation of COPD. Further, the serum FABP-4 and IL-6 levels were significantly higher in patients with stages III-IV COPD than in stages I-II according to GOLD grade. There was a negative correlation between serum FABP-4 levels and SpO2 as well as with FEV1. On the other hand, there was a positive correlation between serum FABP-4 levels and IL-6 as well as with severity of COPD based on GOLD grade. 

The main symptom of COPD, especially its acute exacerbation phase, is the high level of inflammation that plays a key role in the development of disease [u291e]. IL-6 is a pro-inflammatory cytokine produced predominantly by a variety of pulmonary cells, including airways epithelial cells, endothelial cells, alveolar macrophage, and fibroblast [u291f]. Indeed, adipokines are modulating mediators of inflammatory and immune responses [u2920]. In addition, our study results showed that there was a strong correlation between FABP-4 and IL-6 levels in patients with COPD, suggesting that adipokines such as FABP-4 may play a critical role in systemic inflammation of COPD patients. Particularly, the results revealed that serum levels of FABP-4 and IL-6 in the AECOPD patients were much higher than those in SCOPD patients. On the other hand, serum levels of FABP-4 in patients with AECOPD based on the severity of the disease in stages III-IV were greater than stages I-II, indicating that FABP-4 plays a major role in the development and persistence of inflammation in patients with COPD. Accordingly, increased levels of FABP-4 in patients with COPD can be indicative of local or systemic inflammation. Although adipose tissue and inflammatory cells such as macrophage are the main secretor sources of FABP-4, it has also been shown that levels of FABP-4 are high in acute lung injury [28]. Of course, it should be noted that the authors did not rule out other pathways other than the inflammatory pathway of FABP-4 in patients with COPD. In patients with rheumatoid arthritis, it has been shown that increased levels of FABP-4 were associated with lipid profiles, independent of the severity of the disease, which indicates the predominant role of FABP-4 in lipid metabolism rather than inflammation pathway [14]. 

A significant negative correlation between SpO2 and FABP-4 levels in our study may reveal another pathophysiological role of FABP-4 in COPD patients. In patients with COPD, one of the pathways activated as a result of hypoxia is endoplasmic reticulum stress response (ER stress). It has been shown that diminished FABP-4 can reduce the apoptosis mediated by ER stress [29]. Therefore, elevated FABP-4 level in patients with COPD, especially in the AECOPD phase may have an effect on ER stress markers and may affect oxygenation changes which required further studies. 

In this study, we additionally found a positive association between serum FABP-4 levels and CAT score plus mMRC scales. It has been reported that physical activity significantly decreases in patients with higher stages of COPD [30]. Another study also proposed that the increase in levels of inflammatory factors such as C-reactive protein, IL-6, and fibrinogen is the main cause for this diminished activity [31]. The rise in serum FABP-4 and IL-6 levels with increasing GOLD grades of COPD could correlate with the effect of systemic inflammation on health status.

Our study had some limitations. Since previous studies have shown that there was sex differences in FABP-4 levels, we did not measure serum FABP-4 levels in female patients with COPD in the acute exacerbation phase. Further, we did not measure other inflammatory markers other than IL-6. Therefore, we are not able to determine the correlation between serum levels of FABP-4 and other inflammatory markers of COPD. Finally, the sample size of our study was small, and such evaluations should be performed with a large sample size. 

In conclusion, in this study, we found that serum FABP-4 and IL-6 levels increases with the enhanced GOLD grades in patients with COPD, markedly in acute exacerbation phase. The increase of FABP-4 had a strong relationship with elevated serum level of IL-6 and severity of hypoxia. Thus, serum FABP-4 levels merit consideration as a useful marker in the pathogenesis of COPD patients.

## Acknowledgement/Disclaimers

This is a report of a database from the study of “Evaluation of serum levels of Fatty Acid Binding Protein-4 and IL-6 in exacerbation phase of COPD patients” that was registered and funded by Research Committee of Ardabil University of Medical Sciences.

## Informed Consent

The study was approved by the Ethics Committee of Ardabil University of Medical Sciences and all of the study participants signed the written consent forms (No. IR.ARUMS.REC.1397.038).
